# Deep Learning Quality Control for RECIST-Oriented Assessment: A Vision Transformer Predicts Inter-Reader Variability of Unidimensional Lesion Measurements on Contrast CT

**DOI:** 10.3390/cancers18152438

**Published:** 2026-07-29

**Authors:** Abdalla Ibrahim, Amer Hammad, Lucy Wang, Hao Yang, Lawrence H. Schwartz, Binsheng Zhao

**Affiliations:** 1 Department of Radiology, Memorial Sloan Kettering Cancer Center, 1275 York Ave, New York, NY 10065, USAzhaob1@mskcc.org (B.Z.); 2 Department of Radiation Oncology, William Beaumont University Hospital-Corewell Health, 3601 W 13 Mile Rd, Royal Oak, MI 48084, USA; 3 The D-Lab, Department of Precision Medicine, GROW—Research Institute for Oncology and Reproduction, Maastricht University, Universiteitsingel 40, 6229 ER Maastricht, The Netherlands; 4 Department of Medicine, College of Medicine, University of Arizona—Tucson, 1501 N Campbell Ave, Tucson, AZ 85724, USA; 5 School of Medicine, New York Medical College, 40 Sunshine Cottage Rd, Valhalla, NY 10595, USA

**Keywords:** RECIST, inter-reader variability, quality control, lung cancer, artificial intelligence, vision transformer

## Abstract

**Background/Objectives:** Inter-reader variation can alter unidimensional tumor measurements used in RECIST-oriented response assessment. Unlike systems that automate segmentation or target selection, this study aimed to predict lesion-specific measurement uncertainty itself. **Methods:** The development cohort comprised 463 lung, liver, and lymph-node lesions from 280 patients in Vol-PACT, with four segmentations per lesion. Inter-reader variability was defined as (maximum longest diameter–minimum longest diameter)/minimum longest diameter. A Swin Transformer was trained to regress this continuous score. A value > 0.20 was used as a pragmatic high-variability alert threshold, with high variability designated as the positive class; this threshold is not equivalent to RECIST progressive disease. Preliminary external evaluation used 21 NSCLC lesions with five reader contours, providing one reader mask at a time. **Results:** The validation and internal test AUCs were 0.74 (95% CI, 0.61–0.85) and 0.89 (95% CI, 0.80–0.95), respectively. On the internal test set, MAE was 0.10 (95% CI, 0.08–0.12), accuracy was 82% (95% CI, 74–90%), sensitivity 72% (95% CI, 59–85%), and specificity for lower-variability lesions was 92% (95% CI, 82–100%). Across the five external readers, AUCs ranged from 0.77 to 0.92; the wide confidence intervals reflect the small external sample. **Conclusions:** This proof-of-concept model may provide a measurement-quality signal that supports target selection or adjudication. Larger multi-institutional studies, patient-level resampling, calibration, ablation testing, and workflow validation are required before clinical use.

## Simple Summary

Oncology imaging often depends on repeated lesion measurements, but different readers may draw lesion boundaries and measure diameters differently. We developed a deep-learning quality-control model that estimates the expected degree of inter-reader variation from a CT image and a lesion mask. The model predicts a continuous variability score; a 20% cutoff is used only as a practical alert threshold and is not equivalent to RECIST progression. In internal testing and preliminary external testing in 21 non-small cell lung cancer lesions, the model showed promising discrimination, although uncertainty was substantial. The approach may help select more stable target lesions or prompt a second review, but larger, multi-institutional and workflow-based validation is required before clinical deployment.

## 1. Introduction

Accurate tumor measurement on serial imaging is central to oncology trials and increasingly influences individual treatment decisions. RECIST 1.1 summarizes target-lesion burden using unidimensional measurements, with longest axial diameter used for non-nodal lesions and short-axis diameter used for lymph nodes. Progressive disease requires a ≥20% increase in the sum of target-lesion diameters relative to nadir together with an absolute increase of at least 5 mm [1,2,3,4]. Measurements close to decision thresholds are therefore vulnerable to reader-dependent differences, particularly when only one or a few lesions dominate the summed burden.

**Figure 1:**
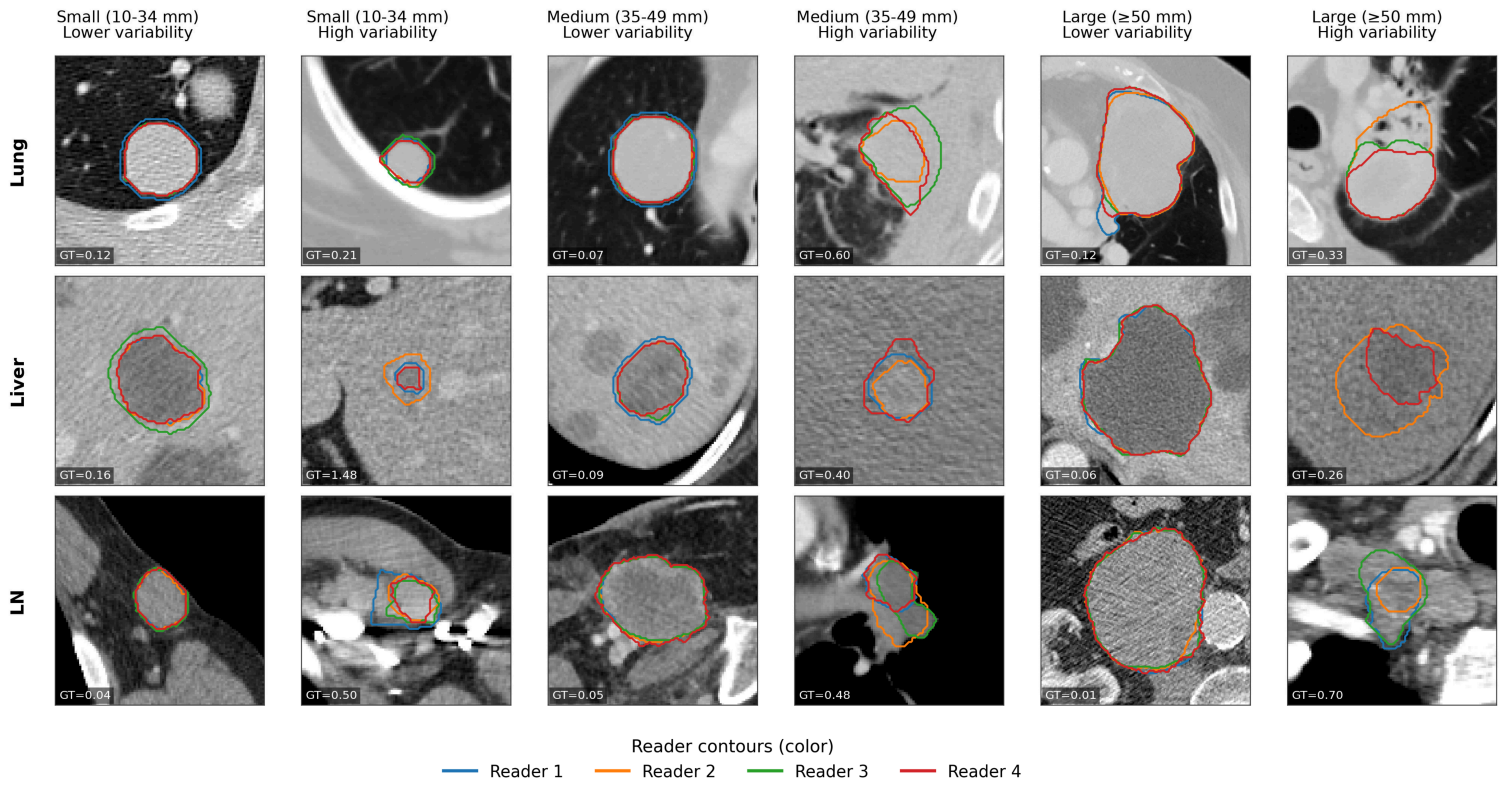
Examples of lower-variability (≤0.20) and high-variability (>0.20) lesions across the development dataset. Reader contours are shown in color; GT denotes the observed continuous inter-reader variability score.

Manual and semi-automated measurements remain affected by lesion selection, boundary interpretation, reader experience, reconstruction settings, contrast phase, and measurement software [5,6,7,8,9,10,11,12,13,14,15,16,17,18,19,20,21]. Even experienced readers can disagree when lesions are small, irregular, heterogeneous, cavitary, poorly conspicuous, adjacent to vessels or atelectasis, pleural-based, hilar, or invasive of the chest wall (Figure 1). Such variation may change an apparent response category even when biological change is limited.

Artificial intelligence has improved lesion detection, segmentation, and automated measurement [22,23,24,25]. However, a technically plausible segmentation does not by itself indicate whether a lesion will be measured consistently by different readers. Automated systems also remain vulnerable to indistinct margins, low lesion-to-background contrast, partial-volume effects, and unusual morphology. This creates a specific unmet need for a quality-control model that estimates measurement uncertainty before a lesion is relied upon for longitudinal assessment.

The need is especially relevant in thoracic oncology. Reliable serial measurements can influence assessment after neoadjuvant chemo-immunotherapy and the timing of surgery [26], evaluation of oligometastatic disease considered for local consolidative treatment [27], distinction of post-treatment change from suspected recurrence after stereotactic body radiotherapy [28], and routine surveillance after lung cancer therapy [29]. In each setting, an uncertain measurement should prompt review within the multidisciplinary tumor board rather than function as an autonomous treatment recommendation.

Measurement confidence may also be useful in lung cancer screening and nodule follow-up. Contemporary management of ground-glass nodules is moving from reflex intervention toward risk-adapted precision surveillance [30]. Within Lung-RADS, a reproducibility warning could potentially identify nodules whose apparent interval growth is close to a category boundary and merits careful remeasurement or comparison with prior examinations [31]. This application remains hypothetical because the present model was not trained on low-dose screening CT or validated for Lung-RADS category assignment.

We therefore developed and preliminarily externally evaluated a vision-transformer model that predicts a continuous lesion-level inter-reader variability score from the CT appearance and a lesion mask. The novel contribution is prediction of measurement uncertainty itself, rather than segmentation, measurement, or target-lesion selection. We hypothesized that image context and contour shape encode lesion-specific features associated with disagreement and that the resulting score could serve as a quality-control signal for RECIST-oriented assessment.

## 2. Materials and Methods

### 2.1. Data Collection

This retrospective secondary analysis used de-identified imaging datasets without links to protected health information. It was determined not to constitute human subjects research and did not require Institutional Review Board oversight. Vol-PACT data were used for model development and internal evaluation, and the publicly available NSCLC Radiomics Interobserver-1 dataset was used for preliminary external evaluation.

The development cohort comprised 463 lung, liver, and lymph-node lesions from 280 patients in Vol-PACT, a partnership established to evaluate quantitative CT biomarkers across ten historical phase III drug trials [32,33]. Four independent semi-automated segmentations and their associated measurements were available for each lesion. Reader-specific specialty, years of experience, and the exact segmentation platform were not retained in the secondary dataset and therefore could not be analyzed.

The preliminary external dataset comprised 21 NSCLC lesions in 21 patients from NSCLC Radiomics Interobserver-1 [34]. Five radiation oncologists manually delineated each lesion in three dimensions. Because of its size, this cohort was treated as a proof-of-concept external evaluation rather than definitive validation.

### 2.2. Diameter Calculation and Variability Endpoint

For each 3D reader segmentation, the axial slice with the largest segmented cross-sectional area was identified, and the longest in-plane diameter was operationalized as the maximum Euclidean distance between boundary points (maximum Feret diameter). The same longest axial diameter was used for lung, liver, and lymph-node lesions. Thus, the nodal analysis does not implement the RECIST 1.1 short-axis rule and should be interpreted as general unidimensional measurement reproducibility rather than strict RECIST nodal assessment. Inter-reader variability was defined as:

(1) $$Variability=\;\frac{{LD}_{max}-{LD}_{min}}{{LD}_{min}}$$

where ${LD}_{max}$ and ${LD}_{min}$ are the maximum and minimum reader-specific diameters for a lesion. The range normalized by the minimum diameter was selected as an intuitive, scale-free measure of worst-case relative disagreement and as a lesion-level continuous target that can be computed from a small number of readers. It is not a conventional RECIST metric and is sensitive to outlying measurements, lesion size, and the number of readers. Coefficient of variation and pairwise differences would provide complementary lesion-level summaries, whereas an intraclass correlation coefficient primarily summarizes reliability across a cohort rather than assigning a score to each lesion.

The model was trained on the continuous variability score to avoid information loss. For secondary discrimination analyses, variability >0.20 was labeled high variability and designated as the positive class; variability ≤0.20 was labeled lower variability. The 0.20 operating point was chosen as a pragmatic quality-control alert inspired by the relative component of the RECIST progression rule. It is not equivalent to progressive disease, which is longitudinal, uses the sum of target-lesion diameters, includes a 5 mm absolute-increase requirement, and applies short-axis measurement to lymph nodes [3,4]. An absolute-difference sensitivity analysis was not performed and is acknowledged as a limitation.

### 2.3. Preprocessing

For model development, a Simultaneous Truth and Performance Level Estimation (STAPLE) consensus mask was derived from the four Vol-PACT segmentations and used as the lesion region of interest. CT volumes and masks were resampled to 1 mm isotropic spacing. The axial slice with the largest lesion area was selected. CT intensities were clipped to a soft-tissue window of −160 to 240 Hounsfield units and linearly scaled to [1]. The CT slice and corresponding binary mask were resized to 224 × 224 and stacked as a two-channel tensor. For single-reader inference, the same preprocessing was applied after substituting an individual reader mask for the STAPLE mask.

### 2.4. Deep-Learning Model Development

The development cohort was split at the patient-level into training (70%; 322 lesions), validation (15%; 64 lesions), and internal test (15%; 77 lesions) sets, with lesion site and variability class balanced as closely as possible. Training augmentation included random flips, small rotations, intensity perturbations, and noise; augmentation was not applied to validation or test data.

A regression model was trained to predict the continuous variability score. Candidate backbones in the Optuna search included Swin Transformer, ResNet, ConvNeXt, and EfficientNet variants implemented in timm. The selected model used the ImageNet-pretrained swin_tiny_patch4_window7_224 backbone, modified for two-channel input and a single continuous output. The architecture search was used for model selection and was not designed as a formal, equally powered ablation comparison.

Optimization used AdamW and Smooth L1 loss. Optuna tuned backbone, learning rate, weight decay, batch size, drop-path rate, and backbone freezing; the best checkpoint was selected by the lowest validation mean absolute error. The final configuration used a learning rate of 2.4 × 10^−5^, weight decay of 0.0055, batch size of 16, drop-path rate of 0.159, and no backbone freezing. Analyses were performed in Python 3.11.

### 2.5. Statistical Analysis

**Figure 2:**
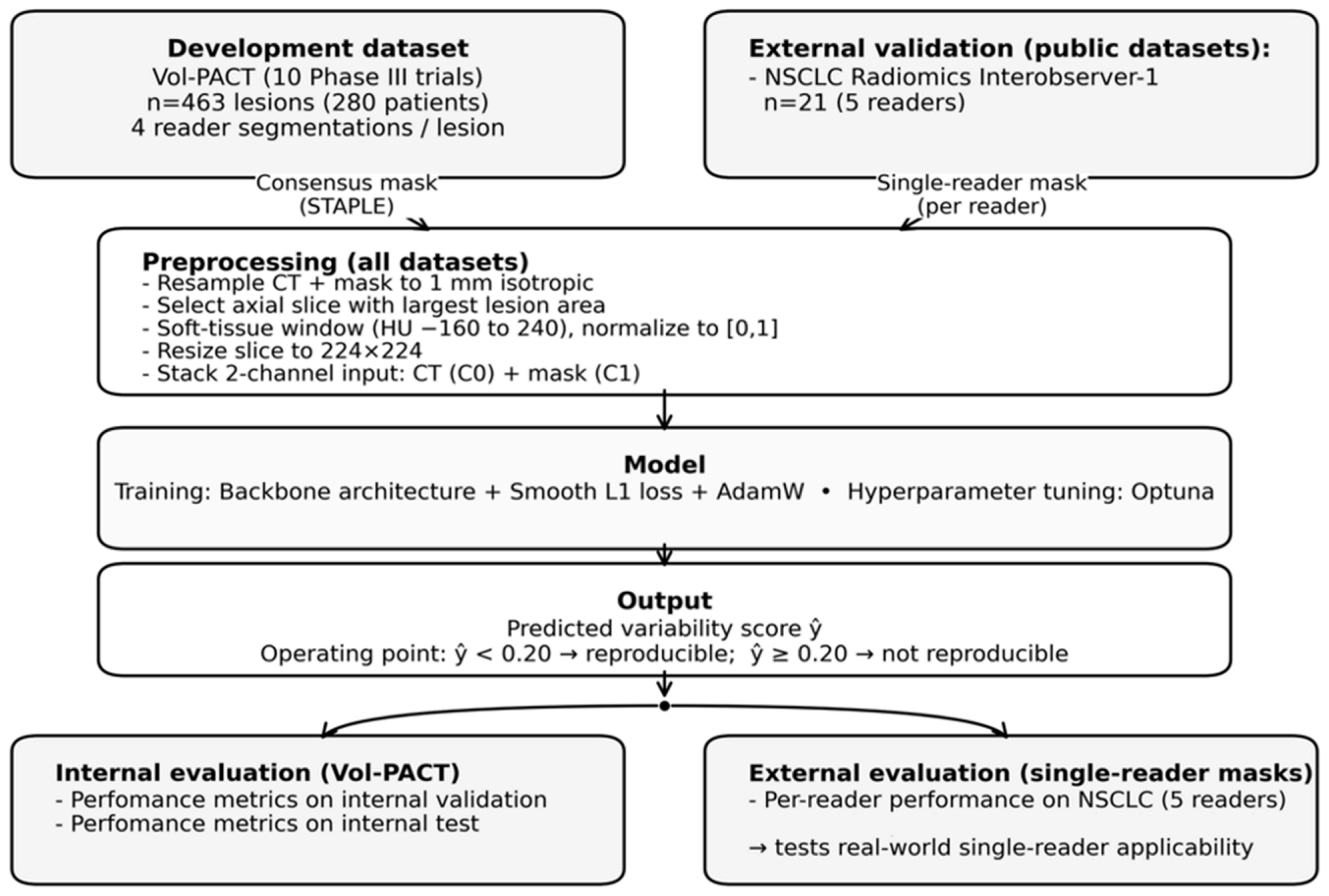
Study workflow for development, internal evaluation, and preliminary external evaluation. High variability (>0.20) is the positive class.

Regression performance was summarized by mean absolute error (MAE). Discrimination between high-variability and lower-variability lesions was summarized by the area under the receiver operating characteristic curve (AUC), using predicted variability as the score and high variability (>0.20) as the positive class. At the prespecified 0.20 operating point, sensitivity refers to high-variability lesions and specificity refers to lower-variability lesions. Confidence intervals for the reported model metrics were generated by nonparametric lesion-level bootstrap within each split; this approach does not fully account for multiple lesions from the same patient and may yield intervals that are too narrow. Exploratory associations of variability class with lesion-size category and anatomical site were assessed with global chi-square tests. To provide simple descriptive references, ordered size category alone (small > medium > large as a risk score) and organ site alone (empirical category risk) were evaluated on the full development cohort; these apparent AUCs are not held-out model comparisons. Single-reader-mask inference was evaluated on the internal test and preliminary external datasets. Figure 2 summarizes the study workflow. No formal calibration slope/intercept, correlation coefficient, R^2^, decision-curve analysis, radiomics baseline, or CT-only/mask-only training ablation was prespecified; the model output should not be interpreted as a calibrated probability of clinical benefit.

## 3. Results

### 3.1. Lesion Characteristics

**Figure 3:**
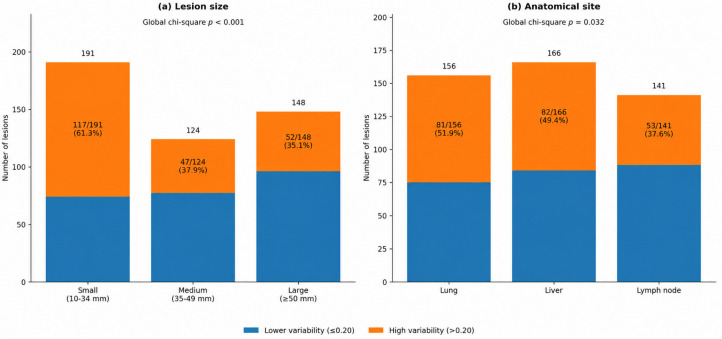
Lesion counts and high-variability proportions according to (**a**) size and (**b**) anatomical site in the development cohort. The displayed *p* values are exploratory global lesion-level chi-square tests and do not adjust for within-patient clustering.

The development cohort included 463 lesions from 280 patients. Of these, 247 (53.3%) had lower variability and 216 (46.7%) had high variability. The cohort comprised 156 lung lesions, 166 liver lesions, and 141 lymph-node lesions. Prespecified size categories included 191 small lesions (10–34 mm), 124 medium lesions (35–49 mm), and 148 large lesions (≥50 mm) (Figure 3). A lesion-level diameter export was not available in the retained analysis archive; therefore, median and interquartile range could not be reconstructed reliably.

The preliminary external cohort comprised 21 NSCLC lesions. Thirteen of 21 lesions (61.9%) met the high-variability definition and eight (38.1%) had lower variability.

### 3.2. Model Performance

**Figure 4:**
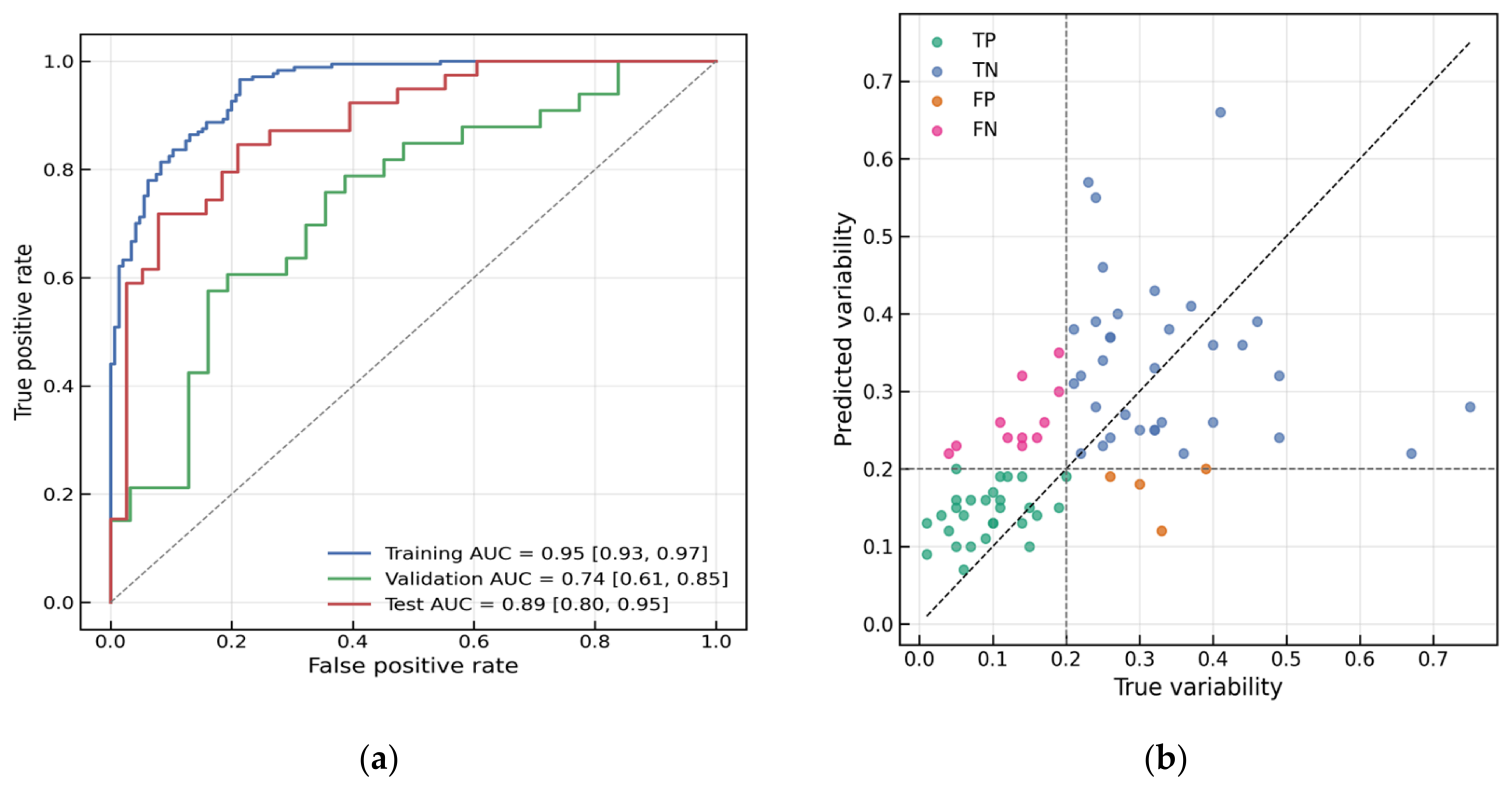
Internal model performance. (**a**) Receiver operating characteristic curves for discrimination of high variability (>0.20), which is the positive class. (**b**) Predicted versus observed continuous variability on the test set; dashed lines mark the 0.20 operating point. TP = true positive, TN = true negative, FP = false positive, FN = false negative. The scatter is descriptive and should not be interpreted as a formal calibration analysis.

The final Swin Transformer was selected by validation MAE. Discrimination for high variability was represented by an AUC of 0.74 (95% CI, 0.61–0.85) on the validation set and 0.89 (95% CI, 0.80–0.95) on the internal test set (Figure 4a). The higher test AUC than validation AUC should not be interpreted as evidence of improved generalization; rather, it underscores sampling variability from a single-fixed split. On the internal test set, MAE was 0.10 (95% CI, 0.08–0.12), accuracy was 82% (95% CI, 74–90%), sensitivity was 72% (95% CI, 59–85%), and specificity 92% (95% CI, 82–100%).

Predicted-versus-observed values on the internal test set are shown in Figure 4b. The scatter demonstrates regression error and threshold-based classification but is not a formal calibration plot. Performance using single-reader masks or the geometric union of reader masks was numerically similar to STAPLE-mask inference, although these comparisons are inference-time stress tests rather than training ablations (Table 1).

### 3.3. Variability According to Lesion Size and Location

High variability was most frequent among small lesions (117/191, 61.3%), compared with medium lesions (47/124, 37.9%) and large lesions (52/148, 35.1%); the global association between size category and variability class was significant (chi-square *p* < 0.001). High variability was also more frequent in lung lesions (81/156, 51.9%) and liver lesions (82/166, 49.4%) than in lymph nodes (53/141, 37.6%), with a global site association of *p* = 0.032.

### 3.4. Preliminary External Validation

**Figure 5:**
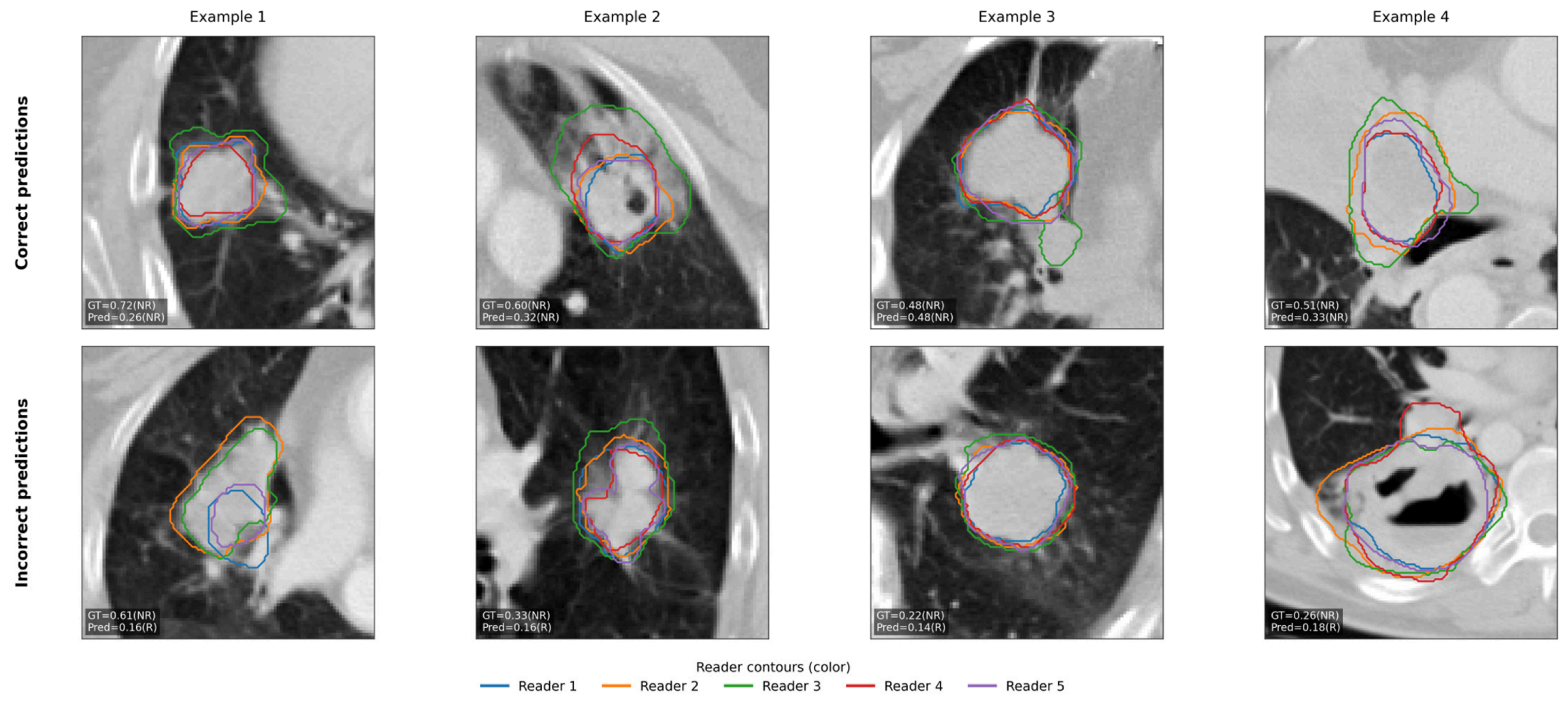
Examples of correct and incorrect high-variability classifications in the preliminary NSCLC Radiomics Interobserver-1 external cohort. Reader contours are shown in color.

To approximate deployment with one available contour, the trained model was applied separately using each external reader mask as the second input channel. The reference variability class for each lesion was calculated from all five reader measurements, whereas each prediction used only one reader mask. Figure 5 shows examples of correctly and incorrectly classified lesions, and the corresponding readers’ segmentations. This evaluates robustness to reader-specific input at inference but does not constitute single-reader-mask training.

Across the five readers, AUCs ranged from 0.77 to 0.92, 60% to 90% sensitivity, 55% to 82% specificity, and accuracy ranged from 67% to 86% (Table 2). Confidence intervals were wide, often extending close to 1.00, because only 21 lesions were available. These findings are therefore preliminary and should not be used to support deployment claims.

## 4. Discussion

This proof-of-concept study developed a Swin Transformer that predicts a continuous lesion-level inter-reader variability score from a CT slice and lesion mask. Internal test discrimination was promising, but the validation AUC was lower and the preliminary external cohort contained only 21 lesions. The model should therefore be viewed as a research quality-control signal rather than a clinically deployable system.

The difference between the validation AUC of 0.74 and test AUC of 0.89 highlights uncertainty from a single-fixed split. The confidence intervals, particularly in the external cohort, further show that plausible performance spans a broad range. Repeated patient-level cross-validation and substantially larger external cohorts are needed to obtain stable estimates and to determine whether performance persists across institutions, scanners, reconstruction kernels, contrast phases, and disease settings.

The model was trained as a regressor, and its continuous output is the primary endpoint. The >0.20 dichotomy is only a practical high-variability alert and is not equivalent to RECIST progressive disease. RECIST progression is based on longitudinal change in the sum of target-lesion diameters, requires an absolute increase of at least 5 mm, and uses short-axis measurements for lymph nodes [3,4]. Consequently, this model cannot determine response category or treatment progression by itself.

The range-based variability metric offers a simple lesion-level estimate of worst-case relative reader spread, but it is sensitive to outliers, lesion size, and reader number. The strong association between small lesion size and high variability confirms this concern. Nevertheless, size-alone showed only modest apparent discrimination in the full cohort, suggesting that additional image and contour information may contribute. Formal held-out comparison with continuous lesion size, coefficient of variation, pairwise differences, and alternative reproducibility targets remains necessary; intraclass correlation should also be reported at the cohort level in future studies.

Thoracic lesion morphology is likely to be an important source of measurement difficulty. Smooth nodules, spiculated primary tumors, cavitary lesions, tumors adjacent to atelectasis, pleural-based masses, hilar lesions, and lesions with chest-wall invasion can create different boundary and axis-selection challenges. The present datasets did not include standardized morphology annotations, so morphology-specific performance could not be quantified. The network may encode some of these visual features, but that possibility should not be interpreted as a substitute for an explicitly annotated morphology analysis.

Potential thoracic applications include baseline target selection and adjudication after neoadjuvant chemo-immunotherapy, where imaging contributes to surgical planning [26]; monitoring oligometastatic disease before and after local consolidative therapy [27]; interpreting residual or enlarging opacity after lung SBRT, where fibrosis can mimic recurrence [28]; and postoperative surveillance after lung cancer treatment [29]. In these settings, the score could flag a measurement for re-review, but decisions regarding surgery, biopsy, salvage therapy, or systemic treatment must remain multidisciplinary and incorporate the full clinical and imaging context.

A related future application is serial nodule follow-up in screening. Precision surveillance of ground-glass nodules aims to balance delayed intervention against unnecessary treatment [30]. A measurement-confidence layer integrated with Lung-RADS could potentially highlight nodules whose apparent growth is close to a category boundary, prompt side-by-side review of prior examinations, or recommend a second measurement [31]. Dedicated validation on low-dose CT, subsolid nodules, and Lung-RADS measurement rules is required before such integration.

Workflow integration also presents practical challenges. A lesion mask must be available from a radiologist or segmentation algorithm; mask generation adds interaction time and may itself be unreliable. A deployable system would require PACS or reporting-system integration, low latency, threshold selection matched to the use case, clear display of uncertainty, monitoring for scanner and population drift, governance for false alerts, and strategies to avoid alert fatigue. Prospective studies should evaluate whether the score changes reader behavior, improves agreement, or merely adds complexity.

Inference with single-reader masks provides a plausible operational pathway because a consensus contour will not exist in routine practice. However, the current single-reader experiments changed the inference input only; the model was still trained with STAPLE masks. They should therefore be interpreted as robustness checks rather than evidence that a single-reader-trained model would perform equivalently.

The use of a STAPLE mask is also a potential source of information leakage because the consensus mask and the variability label are both derived from the same reader contours. A consensus contour may encode the spatial consequences of disagreement even after individual contours are collapsed. The external single-reader-mask results partially reduce, but do not eliminate, this concern. Definitive evaluation requires CT-only, mask-only, CT-plus-mask, single-reader-mask-trained, and independently generated-mask models, ideally with labels constructed from a separate set of readers.

The proposed approach complements prior work rather than replacing it. Studies have documented substantial reader variation in RECIST measurements and target selection [5,35,36]. Machine-learning systems that reproduce reader-specific target selection address which lesion is chosen, whereas the present model asks how consistently a chosen lesion may be measured. Future systems could combine lesion detection, target selection, segmentation, and uncertainty estimation, but each component should be independently validated.

Dataset limitations substantially constrain generalizability. The development cohort was derived from historical trials and included only lung, liver, and lymph-node lesions. Acquisition parameters, patient demographics, treatment context, and reader characteristics were incompletely available. The external cohort was single-disease, small, and not sufficient to establish performance across institutions or lesion phenotypes.

Statistical limitations also remain. Confidence intervals were generated by lesion-level rather than patient-level bootstrap and may be too narrow when patients contributed multiple lesions. Formal calibration, correlation, R^2^, decision-curve analysis, and clinically defined net-benefit thresholds were not available. The output is a continuous variability estimate, not a calibrated probability, and Figure 4b should be read as a descriptive regression plot.

Technical limitations include use of a single axial slice, dependence on the supplied segmentation, absence of an absolute-difference sensitivity analysis, and longest-axis rather than short-axis measurement for lymph nodes. Healthy tissue inclusion, missed tumor extensions or spiculations, partial-volume effects, or inconsistent plane selection may alter the score. The model was not evaluated with morphology labels, radiomics baselines, CT-only or mask-only training, or equally powered alternative-backbone ablations.

Future work should prioritize larger multi-institutional cohorts, repeated patient-level validation, strict RECIST nodal measurements, paired relative-and-absolute variability endpoints, independent-reader labels, morphology annotation, calibration and decision-curve analysis, and prospective workflow testing. Three-dimensional and segmentation-free variants may further reduce sensitivity to slice and mask selection. Only after these steps should the model be considered for RECIST quality control, Lung-RADS follow-up support, or personalized surveillance pathways.

## 5. Conclusions

In conclusion, a vision-transformer model showed proof-of-concept ability to estimate lesion-level inter-reader variability in unidimensional CT measurements. The continuous score may eventually support target selection or second review, but the current evidence is preliminary and does not establish RECIST progression, calibrated clinical utility, or readiness for deployment.

## Figures and Tables

**Table 1:** Model performance on the internal validation and test sets.

Split	AUC (95% CI)	MAE (95% CI)	Sensitivity (95% CI)	Specificity (95% CI)	Accuracy (95% CI)
Validation	0.74 (0.61–0.85)	0.12 (0.09–0.15)	61 (45–76)	71 (55–87)	66 (55–77)
Test					
STAPLE	0.89 (0.78–0.95)	0.10 (0.08–0.12)	72 (59–85)	92 (82–100)	82 (74–90)
Reader 1	0.87 (0.78–0.94)	0.11 (0.09–0.13)	72 (56–85)	89 (79–97)	81 (71–90)
Reader 2	0.85 (0.77–0.93)	0.12 (0.10–0.14)	64 (49–79)	89 (79–97)	77 (68–84)
Reader 3	0.85 (0.75–0.93)	0.11 (0.09–0.13)	67 (50–83)	87 (74–97)	77 (68–86)
Reader 4	0.87 (0.79–0.94)	0.11 (0.09–0.13)	67 (51–82)	89 (79–97)	78 (69–86)
Union	0.84 (0.74–0.92)	0.11 (0.09–0.14)	73 (59–86)	84 (71–95)	79 (69–87)

Note: High variability (>0.20) is the positive class. Sensitivity, specificity, and accuracy are percentages. Confidence intervals were estimated by lesion-level bootstrap.

**Table 2:** Per-reader performance in the preliminary NSCLC Radiomics Interobserver-1 external cohort.

Reader	AUC (95% CI)	MAE (95% CI)	Sensitivity (95% CI)	Specificity (95% CI)	Accuracy (95% CI)
1	0.91 (0.75–1.00)	0.12 (0.08–0.17)	90 (70–100)	64 (36–91)	76 (57–90)
2	0.92 (0.76–1.00)	0.10 (0.06–0.15)	90 (70–100)	82 (55–100)	86 (71–100)
3	0.83 (0.63–0.98)	0.11 (0.07–0.14)	60 (30–90)	82 (55–100)	71 (52–90)
4	0.77 (0.55–0.96)	0.10 (0.05–0.16)	80 (50–100)	64 (36–91)	71 (52–90)
5	0.81 (0.76–1.00)	0.12 (0.08–0.18)	80 (50–100)	55 (27–82)	67 (48–86)

Note: High variability (>0.20) is the positive class. Sensitivity, specificity, and accuracy are percentages. The cohort contains 21 lesions; confidence intervals are therefore imprecise.

## Data Availability

The NSCLC Radiomics Interobserver-1 dataset is publicly available through The Cancer Imaging Archive. The Vol-PACT development data are not publicly available. Please contact Binsheng Zhao (zhaob1@mskcc.org).
